# Supplementary material for: Potential of the Red Alga Dixoniella grisea for the Production of Additives for Lubricants
Source: Plants (Basel). 2021 Sep 4;10(9):1836. doi: 10.3390/plants10091836 (PMC8465309; doi:10.3390/plants10091836)
Supplement: Supplementary file 1 [file plants-10-01836-s001.zip › plants-1355003-supplementary.pdf]

Supplementary Material:

1. *Validation of analytical procedures*

Calibration was done with two sugar standard mixtures: the 7-Mix contained Ara, Rib, Xyl, Fru, Man, Gal, Glc and the NEW-Mix contained Rha, Fuc, Gul, GlcUA, GalUA, GlcNA and Ery as internal standard. All standards mixtures were diluted in methanol, concentrations ranged between 5 to 100 mg L^{-1} each. Prior to derivatization, all mixtures in all concentrations were kept in portions per vial. All standards were dried and stored in a deep freezer until use. When used Ery and oleic acid (OA) as internal standards, the methanol was evaporated at room temperature after addition to the sample.

All monosaccharides were detected with two TMSE-signals; the first shows the cyclic form, the second correspond to the open chain form of each sugar standard. In these, an adequate separation of the signals could be observed. Most of the sugars were detected in a time range from 12.5 min to 20.05 min (Table SI 1). Erythritol (10.2 min) as internal standard was the first compound to measure, followed by the pentose arabinose at 12.55 min (ring form) and 13.21 min (as open chain). Glucose was the last monosaccharide at 18.23 min (ring) and 20.05 min (chain). The single sugars mannose (main signal) and fructose (2nd signal) were determined together, a separation of the peak of the GC-MSD was not obvious; the same happened with the second signal of mannose and main signal of galactose. The factors are 3:1 for the first pair and 1:3 for the second pair. The three sugar acids galacturonic acid, glucuronic acid, and N-acetyl-glucosamine eluted last at retention times of 19.9/20.69 min, 19.5/22.19 min and 22.25/22.47 min, respectively. The oleic acid as TMSE-derivative was detected at 24.33 min.

Table SI 1. Retention time and area precision (given as RSD % (see 4.8)) for 13 sugars (in a concentration range from 0.01 – 0.1 g L^{-1}) separated as TMSE-derivatives via GC-MSD on a CS FS Supreme-5ms column (n = 7). Oleic acid was measured with both methods to ensure that silylation is of equal quality.

		Main Signal (TIC)		SIM	LOD	LOQ	R^2	2nd Signal (TIC)		SIM	LOD	LOQ	R^2
	**Substance**	**RT RSD [%]**	**Area RSD [%]**	**Area RSD [%]**	**[g L^{-1}]**	**[g L^{-1}]**	**[0.01-0.1 g L^{-1}]**	**RT RSD [%]**	**Area RSD [%]**	**Area RSD [%]**	**[g L^{-1}]**	**[g L^{-1}]**	**[0.01-0.1 g L^{-1}]**
0	Erythritol	1.58	3.88		0.001	0.005	0.9851						
1	Arabinose	5.88	3.88		0.001	0.005	0.9286	6.06	6.35		0.005	0.01	0.9854
2	Ribose	5.9	5.06		0.001	0.005	0.9715	5.78	7.22		0.005	0.01	0.9447
3	Xylose	5.28	4.19		0.001	0.005	0.947	5.52	5.54		0.005	0.01	0.9697
4	Mannose	5.16	5.26		0.001	0.005	0.9943	4.09	4.44		0.005	0.01	0.9857
5	Fructose	5.08	1.09		0.005	0.01	0.9951	5.07	5.26		0.03	0.06	0.9795
6	Galactose	4.75	4.65		0.001	0.005	0.9654	4.9	4.81		0.005	0.01	0.9709
7	Glucose	4.49	4.44		0.001	0.005	0.9697	4.79	5.73		0.005	0.01	0.9595
8	Rhamnose	1.35	2.88		0.001	0.005	0.9919	1.47	2.88		0.01	0.02	0.9905
9	Fucose	1.25	4.34		0.001	0.005	0.9922	1.51	7.67		0.01	0.02	0.9913
10	Gulose	1.11	7.36		0.005	0.01	0.9829	1.26	7.6		0.02	0.04	0.9705
11	Galacturonic acid	2.95	9.43	1.33*	0.01*	0.02*	0.9634	1.16		5.26*	0.03*	0.03*	

12	Glucuronic acid	2.17	7.67	2.01*	0.02*	0.04*	0.9823	1.54		0.31*	0.03*	0.05*	
13	N-Acetyl-Glucoseamine	1.99	9.77	3.53§	0.02§	0.04§	0.9724	0.31		2.31§	0.02§	0.05§	0.9941
14	Oleic Acid as TMSE	0.04	3.82		0.001	0.005	0.9974						
	Oleic Acid as FAME	0.04	1.82		0.0005	0.001	0.9986						

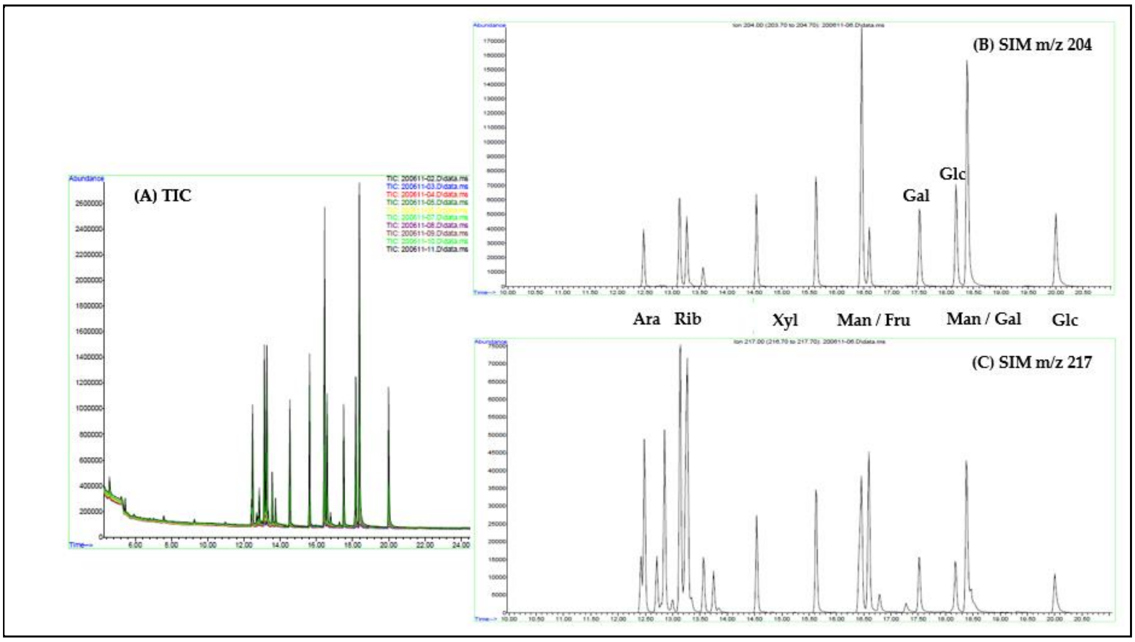

Figure SI 1. Chromatogram of: (A) Seven Monosaccharide standards Ara, Rib, Xyl, Man, Fru, Gal, and Glc as their TMSE-derivatives (TIC) at a concentration of 50 mg L^{-1} analyzed on an Agilent GC-MSD-System with CS FS Supreme-5ms column (n = 7). For verification of the pentoses and hexoses the SIM-Modes (B) m/z 204 and (C) m/z 217 were used.

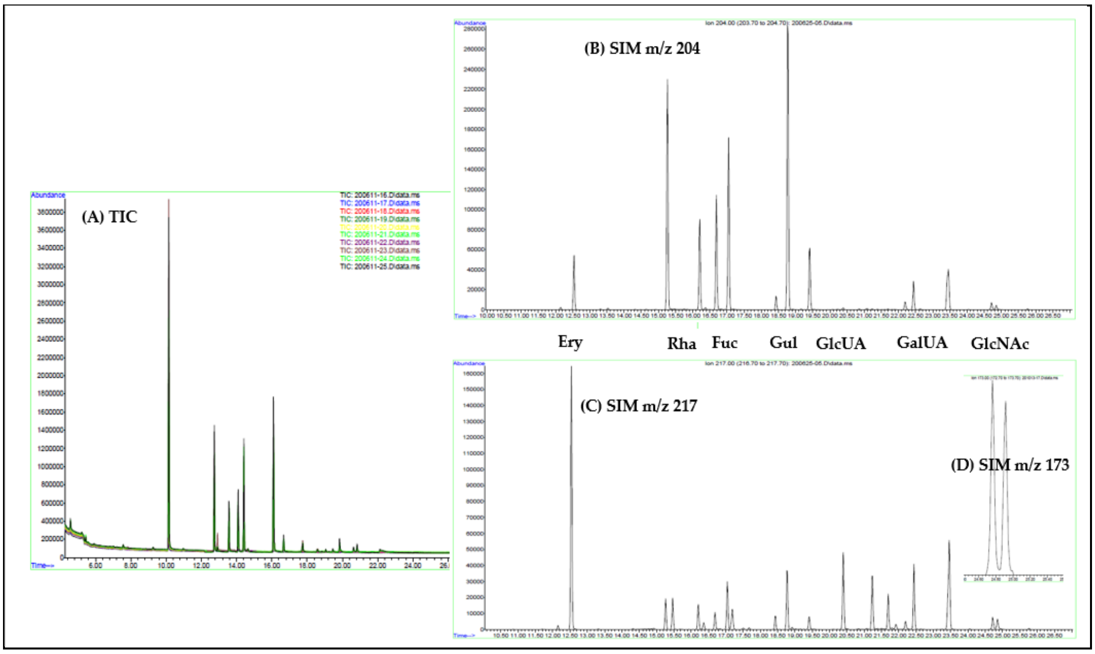

Figure SI 2. Chromatogram of: (A) Seven Monosaccharide standards Ery (as IS), Rha, Fuc, Gul, GlcUA, GalUA, and GlcNAc as their TMSE-derivatives (TIC) at a concentration of 50 mg L^{-1} analyzed on an Agilent GC-MSD-System with CS FS Supreme-5ms column (n = 7). For verification of the pentoses, hexoses, and sugar acids the SIM-Modes (B) m/z 204 and (C) m/z 217 were used.

In comparison with the single monosaccharides in the standard mixtures, algae samples did not always correspond well to the single sugar standard mixes (Figures SI 1 vs. SI 2). The higher signal intensity fitted to either a galactose-oxime as hexa-TMSE derivative or the corresponding glucose-oxime, with additional smaller contributions from palmitic and stearic acid (C16:0 and C18:0, respectively) (Figure SI 3). To allow the summary of all obtained results, only the major signals were included in the further analysis.

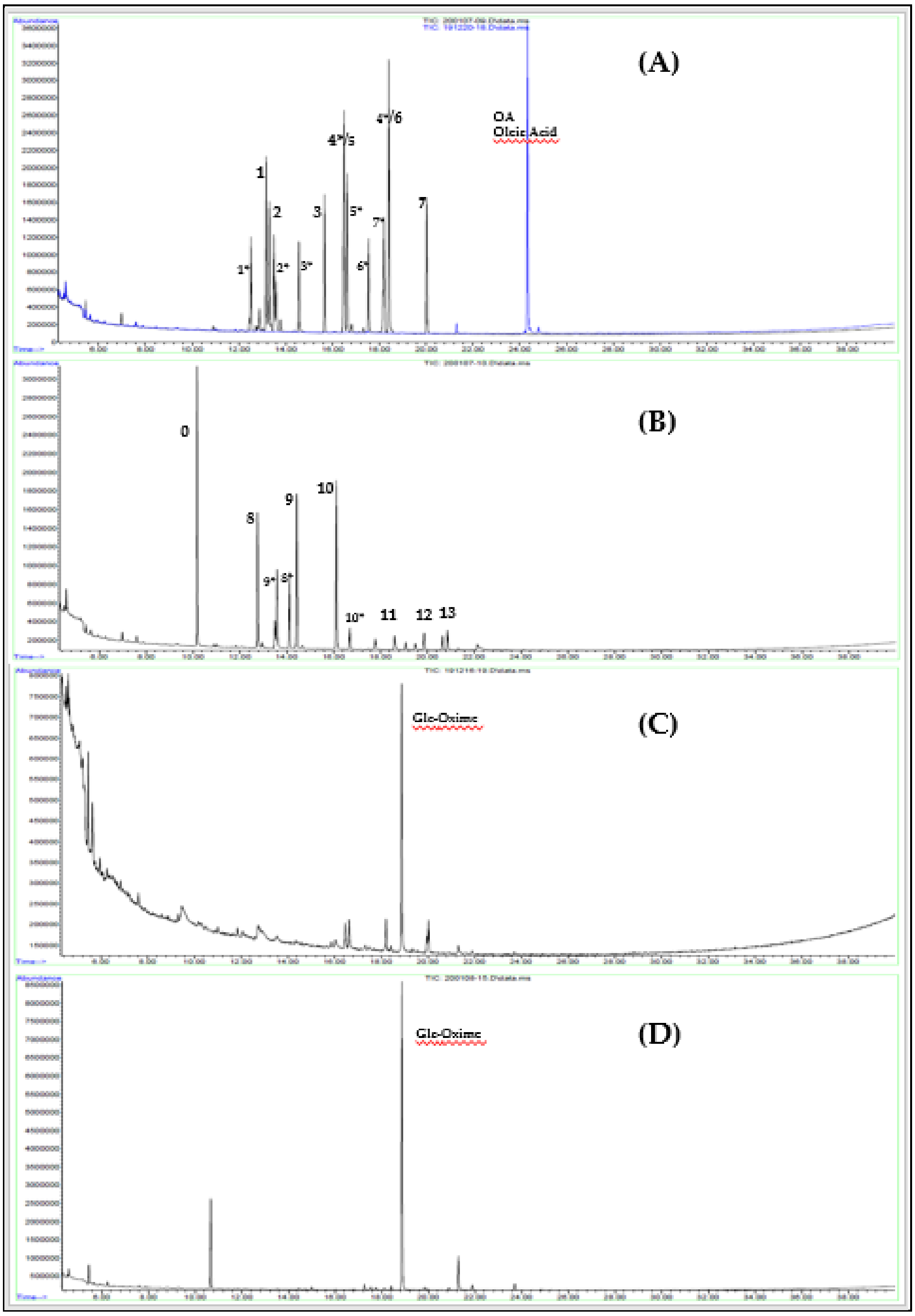

Figure SI 3. Chromatogram of: (A) internal standard of oleic acid as TMSE at a concentration of 50 mg L^{-1} as TIC. and seven sugar standards as their TMSE-derivatives at a concentration of 50 mg L^{-1} as TIC. The numbers correspond to main signals: (1) Ara, (2) Rib, (3) Xyl, (4) Man, (5) Fru, (6) Gal, and (7) Glc. Numbers with (*) marked the lower second signals. (B) Seven sugar standards as their TMSE-derivatives at a concentration of 50 mg L^{-1} as TIC. The numbers correspond to main signals: (0) Ery as IS, (8) Rha, (9) Fuc, (10) Gul, (11) GlcUA, (12) GalUA, and (13) GlcNAc. Numbers with (*) marked the lower second signals. (C) Sample of *D. grisea* EPS collected after precipitation with 2-propanol, after freeze drying as TMSE-derivative.

(D) Sample of *D. grisea* pellet collected after precipitation with 2-propanol, centrifugation, and freeze drying as TMSE-derivative. All samples were analyzed on an Agilent GC-MSD-System with CS FS Supreme-5ms column (n = 7).

For primary amino acid internal standard (ISTD) stock solutions, 58.58 mg norvaline was weighed into a 50 mL volumetric flask. For secondary amino acids, 44.54 mg sarcosine was weighed into the same 50 mL flask. This flask was filled halfway with 0.1 N HCl, and shaken or sonicated until dissolved, then filled to mark with water for a final concentration of 10 nmol each amino acid µL^{-1} (standard sensitivity). For high-sensitivity external standard stock solution, 5 mL of standard-sensitivity solution was diluted with 45 mL of water, and stored at 4 °C.

For calibration curves, five amino acid standards in the concentration range of 100, 250, 500, 750, 1000 pmol µl^{-1}, purchased from Agilent (p/n 5061-3330 - 5061-3334). This five-point-calibrations were assayed in triplicate for sensitivity analysis. The amino acids were identified in the samples with the comparison of the retention time of the standards. As ISTDs norvaline and sarcosine (10 nmol µl^{-1} in 0.1 M HCl) 100 µl were added to each diluted standard, and stored at 4 °C.

In the resulting chromatograms, 23 amino acid standards were detected in a time of 1.9 to 15.5 min as their OPA resp. FMOC-derivatives at a concentration of 100 to 1250 pmol µL^{-1}. The primary amino acids and norvaline as internal standard as their OPA-derivatives were measured at 338 nm: aspartic acid, glutamic acid, asparagine, serine, glutamine, histidine, glycine, threonine, arginine, alanine, tyrosine, cysteine, valine, methionine, norvaline (internal standard, 10.2 min), tryptophane, phenylalanine, isoleucine, leucine, lysine, at 338 min. The two secondary amino acids hydroxyproline and proline, and sarcosine as additional internal standard (IS, 15.0 min) were detected as their FMOC derivatives at 262 nm. Table SI 2 shows the observed LOD and LOQ values of the amino acids. The LOD and LOQ were approximately 10 pmol and 25 pmol by using UV detection. The calibration curves were prepared with standard solutions containing 23 amino acid standards at a concentration of 1 nMol mL^{-1} each.

Table SI 2. LOD, LOQ, retention time and area precision (given as RSD % (see 4.8)) for 23 amino acid standards (in a concentration range from 100 – 1.000 pmol µL^{-1}) separated as OPA and FMOC derivatives via HPLC-DAD on an Agilent AdvanceBio AAA 4.6 x 100 mm, 2.7 µm column, using the amino acid method (n = 5). IS: internal standard substance.

			AREA	RSD	[pmol µl^{-1}]	
**No.**	**Name**	**RT [min]**	**[%]**	**R^2**	**LOD**	**LOQ**
1	Aspartic Acid	1.4	8.47	0.9819	50	100
2	Glutamic Acid	2.1	7.90	0.9648	50	100
3	Asparagine	2.5	5.85	0.9497	50	100
4	Serine	4.3	9.84	0.9960	100	150
5	Glutamine	4.8	8.55	0.9969	50	100
6	Histidine	5.6	8.23	0.9955	50	100

7	Glycine	5.9	8.30	0.9587	50	100
8	Threonine	6.0	9.53	0.9881	50	100
9	Arginine	7.1	7.70	0.9939	50	100
10	Alanine	7.3	7.78	0.9882	50	100
11	Tyrosine	8.4	7.81	0.9958	50	100
12	Cysteine	9.0	9.07	0.9934	50	100
13	Valine	9.6	8.86	0.9807	50	100
14	Methionine	10.0	3.57	0.9981	50	100
15	NORVALINE **IS**	10.4	2.57	0.9973	50	100
16	Tryptophane	10.7	6.57	0.9943	50	100
17	Phenylalanine	10.9	2.19	0.9981	50	100
18	Isoleucine	11.1	4.78	0.9916	50	100
19	Leucine	11.7	5.28	0.9803	50	100
20	Lysine	13.0	7.25	0.9980	50	100
21	Hydroxyproline	14.4	10.30	0.9854	100	150
22	SARCOSINE **IS**	15.0	4.36	0.9958	100	150
23	Proline	15.5	11.74	0.9891	100	150

For proteins, an adequate separation of the signals can be observed for the amino acid standards, as well as freeze dried samples of *D. grisea* EPS and pellet collected after precipitation with 2-propanol as their OPA- and FMOC-derivative (Figure SI 4). The chromatograms for standard amino acids and extracted samples fitted well. The only exception was the signal at 10.6 min for the amino acid lysine, so that this amino acid was corrected in the further analysis.

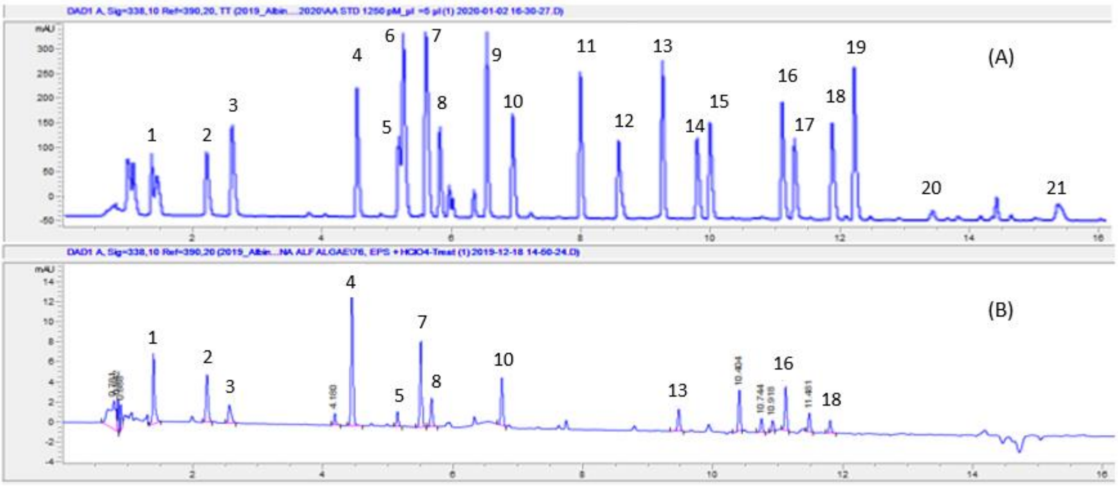

Figure SI 4. Separation of amino acid standard (1 nmol) on an Agilent AdvanceBio AAA 4.6 x 100 mm, 2.7 µm column, using the amino acid method. Chromatogram of: (A) 23 amino acid standards as their OPA resp. FMOC-derivatives at a concentration of 1250 pmol µl^{-1} at 338 nm. The numbers correspond to: (1) aspartic acid, (2) glutamic acid, (3) asparagine, (4) serine, (5) glutamine, (6) histidine, (7) glycine, (8) threonine, (9) arginine, (10) alanine, (11) tyrosine, (12) cysteine, (13) valine, (14) methionine, (15) tryptophane, (16) phenylalanine, (17) isoleucine, (18) leucine, (19) lysine, (20) hydroxyproline, and (21) proline. (B) Sample of EPS of *D. grisea*

2. Overview on the compositions of the test media used for ecotoxicological tests

Table SI 3. Artificial soil pore water E: test medium for *E. crypticus*

Chemical	Amount [mg L^{-1}]
Sodium bicarbonate (7.5 ml)	64.5
Potassium chloride (8 ml)	5.92
Calcium chloride · $2H_2O$ (10 ml)	294
Magnesium sulfate (10 ml)	123

Table SI 4. Artificial soil pore water C: test t medium for *F. candida*

Chemical	Amount [g L^{-1}]
Sea salt*	0.5

Potassium dihydroxyphosphate	0.3

* TropicMarin ProReef, Art.-Nr. 10581

Table SI 5. Composition of test medium for *A. globiformis*

Chemical	Amount [gL^{-1}]
Peptone from casein	3.33
Yeast extract	1.67
Sodium chloride	1.67
D(+) glucose	1.67

Table SI 6. Resources of the chemicals to prepare the ecotoxicological test media

**Chemical**	**Resource**
Ammonium vanadate	Sigma-Aldrich GmbH, Steinheim, Germany
Boric acid	Sigma-Aldrich GmbH, Steinheim, Germany
Calcium chloride · $2H_2O$	VWR Chemicals International bvbs Geeldenaaksebaan, Leuven, Belgium
Cobalt chloride · 6 H_2O	Merck KgaA, Darmstadt, Germany
Copper chloride · $2H_2O$	Sigma-Aldrich GmbH, Steinheim, Germany
D(+) glucose	Carl-Roth GmbH und Co.KG, Karlsruhe, Germany
Iron sulfate · $7H_2O$	Merck KgaA, Darmstadt, Germany
Lithium chloride	Sigma-Aldrich GmbH, Steinheim, Germany
Manganese chloride · 4 H_2O	Riedel-de Haen by Sigma-Aldrich GmbH, Seelze, Germany
Magnesium sulfate · 7 H_2O	Chemsolute Th. Geyer GmbH & Ko.KG, Renningen, Germany

Peptone from casein	Sigma-Aldrich GmbH, Steinheim, Germany
Potassium chloride	Carl-Roth GmbH und Co.KG, Karlsruhe, Germany
Potassium iodide	Sigma-Aldrich GmbH, Steinheim, Germany
di-Potassium hydrogenphosphate	Carl-Roth GmbH und Co.KG, Karlsruhe, Germany
Potassium dihydrogenphosphate	Carl-Roth GmbH und Co.KG, Karlsruhe, Germany
Sodium bromide	Sigma-Aldrich GmbH, Steinheim, Germany
Rubidium chloride	Sigma-Aldrich GmbH, Steinheim, Germany
Sodium bicarbonate	VWR Chemicals International bvbs Geeldenaaksebaan, Leuven, Belgium
Sodium chloride	VWR Chemicals International bvbs Geeldenaaksebaan, Leuven, Belgium
SodiumEDTA · $2H_2O$	Merck KgaA, Darmstadt, Germany
Sodium metasilicate · 5 H_2O	Sigma-Aldrich GmbH, Steinheim, Germany
Sodium molybdate · $2H_2O$	Merck KgaA, Darmstadt, Germany
Sodium nitrate	Carl-Roth GmbH und Co.KG, Karlsruhe, Germany
Sodium selenite	Sigma-Aldrich GmbH, Steinheim, Germany
Strontium chloride · 6 H_2O	Sigma-Aldrich GmbH, Steinheim, Germany
Yeast extract	Merck KgaA, Darmstadt, Germany
Zinc chloride	Riedel-de Haen by Sigma-Aldrich GmbH, Seelze, Germany
